# Supplementary material for: The social and environmental complexities of extracting energy transition metals
Source: Nat Commun. 2020 Sep 24;11:4823. doi: 10.1038/s41467-020-18661-9 (PMC7519138; doi:10.1038/s41467-020-18661-9)
Supplement: Supplementary file 1 — Supplementary Information [file 41467_2020_18661_MOESM1_ESM.pdf]

Supplementary information

The social and environmental complexities of extracting energy transition metals

Lèbre *et al.*

Supplementary tables

Supplementary Table 1: List of data sources used to build Figures 1c and 1d

Authors	Title
Davidsson and Höök (2017)	Material requirements and availability for multi-terawatt deployment of photovoltaics
de Koning, Kleijn et al. (2018)	Metal supply constraints for a low-carbon economy?
Deetman , Pauliuk et al. (2018)	Scenarios for Demand Growth of Metals in Electricity Generation Technologies, Cars, and Electronic Appliances
Elshkaki and Graedel (2015)	Solar cell metals and their hosts: A tale of oversupply and undersupply
Giurco, Dominish et al. (2019)	Requirements for Minerals and Metals for 100% Renewable Scenarios
Harvey (2018)	Resource implications of alternative strategies for achieving zero greenhouse gas emissions from light-duty vehicles by 2060
Hertwich, Gibon et al. (2015)	Integrated life-cycle assessment of electricity-supply scenarios confirms global environmental benefit of low-carbon technologies
Hund, La Porta et al. (2020)	Minerals for Climate Action: The Mineral Intensity of the Clean Energy Transition
Li and Adachi (2019)	Evaluation of long-term silver supply shortage for c-Si PV under different technological scenarios
Månberger and Stenqvist (2018)	Global metal flows in the renewable energy transition: Exploring the effects of substitutes, technological mix and development
Pehlken, Albach et al. (2017)	Is there a resource constraint related to lithium ion batteries in cars?
Rasmussen, Wenzel et al. (2019)	Platinum Demand and Potential Bottlenecks in the Global Green Transition: A Dynamic Material Flow Analysis
Valero, Valero et al. (2018)	Material bottlenecks in the future development of green technologies
Watari, Nansai et al. (2020)	Review of critical metal dynamics to 2050 for 48 elements
Watari, McLellan et al. (2019)	Total Material Requirement for the Global Energy Transition to 2050: A focus on transport and electricity
Watari, McLellan et al. (2018)	Analysis of Potential for Critical Metal Resource Constraints in the International Energy Agency's Long-Term Low-Carbon Energy Scenarios
Ziemann, Müller et al. (2018)	Modeling the potential impact of lithium recycling from EV batteries on lithium demand: A dynamic MFA approach

Supplementary Table 2: Commodity coverage in the S&P Global Market Intelligence database (S&P database)

Commodity	Percentage of production covered by S&P database	Estimated global production (average of 2018 and 2019)*
Platinum	100%	0.185 kt
Uranium Oxide	99%	63 kt***
Iron	99%	1,485,000 kt
Nickel	97%	2,550 kt
Copper	96%	20,200 kt
Lithium	95%	86 kt
Silver	95%	27 kt
Cobalt	84%	144 kt
Gold	81%	3.3 kt
Zinc	77%	12,750 kt
Bauxite	72%	348,500 kt
Molybdenum	70%	294 kt
Manganese	59%	18,950 kt
Lead	59%	4,530 kt
Rare Earths**	Not Available	200 kt
Heavy Mineral Sands	N.A.	N.A.
Tin	N.A.	314 kt
Tungsten	N.A.	83 kt

*Source: USGS (2020), unless stated otherwise.

** In the analysis, lanthanides, scandium and yttrium are grouped together as rare earths. The coverage level for rare earths production is not provided by the S&P database, which means there is a higher uncertainty around ESG results for rare earths.

*** Year 2016, S&P database estimate.

Supplementary Table 3: Correlations across the seven ESG dimensions

	Waste	*Water*	*Conservation*	*Communities*	*Land Uses*	*Social Vulnerability*	*Governance*
Waste	1						
Water	-0.069	1					
Conservation	0.210	-0.036	1				
Communities	0.029	-0.063	0.421	1			
Land Uses	-0.014	0.158	0.167	0.125	1		
Social Vulnerability	-0.059	-0.104	0.553	0.419	0.072	1	
Governance	-0.005	-0.057	0.499	0.442	0.098	0.808	1

Supplementary Table 4: Correlations across the 24 variables

	Control of Corruption	Government Effectiveness	Political Stability No Violence	Regulatory Quality	Rule of Law	Voice and Accountability	Human Development Index	Gini coefficient	Total Dependency Ratio	Earthquakes	Terrain Ruggedness Index	Precipitations	Wind	Cyclones	Distance to biodiversity areas	Combined Species Richness	Baseline Water Stress	Annual Water Variability	Indigenous Peoples Lands	Population Density (100 km)	Population Density (1 km)	Pasture Lands	Croplands	Forest lands
CC	1																							
GE	0.924	1																						
PS	0.884	0.837	1																					
RQ	0.911	0.931	0.830	1																				
RL	0.980	0.945	0.890	0.946	1																			
VA	0.870	0.768	0.822	0.873	0.880	1																		
HDI	0.785	0.904	0.764	0.858	0.830	0.699	1																	
Gini	-0.275	-0.310	-0.391	-0.273	-0.296	-0.155	-0.348	1																
TDR	-0.013	-0.019	-0.023	-0.014	-0.012	-0.009	-0.021	0.014	1															
E	-0.021	-0.002	-0.026	-0.012	-0.015	-0.040	0.016	0.040	-0.039	1														
TRI	-0.190	-0.144	-0.212	-0.123	-0.193	-0.167	-0.098	0.120	0.020	0.033	1													
P	-0.188	-0.240	-0.178	-0.201	-0.206	-0.116	-0.266	0.076	0.023	0.031	0.175	1												
W	0.007	0.016	0.002	0.017	0.007	0.010	0.013	0.005	0.003	0.152	0.022	0.310	1											
C	0.010	-0.002	-0.028	0.017	-0.004	0.028	-0.002	-0.025	-0.004	-0.061	-0.012	0.121	-0.072	1										
DBA	-0.329	-0.342	-0.324	-0.253	-0.335	-0.211	-0.272	0.235	0.017	-0.048	0.301	0.253	0.004	0.075	1									
CSR	-0.464	-0.545	-0.472	-0.487	-0.463	-0.339	-0.572	0.513	0.022	0.077	0.117	0.420	0.042	-0.033	0.278	1								
BWS	-0.030	-0.011	-0.081	-0.029	-0.045	-0.041	0.087	0.085	0.005	0.097	0.022	0.011	-0.001	0.007	0.022	0.071	1							
AWV	0.012	0.051	-0.033	0.044	0.024	-0.006	0.079	0.010	0.001	0.179	-0.012	-0.113	0.063	-0.116	-0.078	0.108	0.485	1						
IPL	-0.062	-0.079	-0.081	-0.088	-0.081	-0.078	-0.091	-0.127	-0.004	0.034	-0.027	-0.003	0.011	0.089	0.006	-0.024	-0.086	-0.020	1					
PD100	-0.316	-0.358	-0.360	-0.375	-0.338	-0.350	-0.437	0.062	0.009	-0.005	0.070	0.150	-0.003	-0.011	0.123	0.231	0.018	0.016	0.056	1				
PD1	-0.153	-0.153	-0.151	-0.187	-0.167	-0.209	-0.171	0.017	0.002	0.018	-0.045	0.120	0.156	-0.016	0.007	0.120	0.007	0.022	-0.026	0.151	1			
PL	-0.041	-0.010	-0.058	-0.014	-0.029	-0.045	0.009	0.047	0.004	0.368	0.029	0.002	0.161	-0.125	-0.051	0.102	0.301	0.449	-0.005	0.021	0.066	1		
CL	-0.041	-0.010	-0.058	-0.014	-0.029	-0.045	0.009	0.047	0.004	0.368	0.029	0.002	0.161	-0.125	-0.051	0.102	0.301	0.449	-0.005	0.021	0.066	-0.065	1	
FL	0.073	0.070	0.136	0.056	0.080	0.102	0.066	-0.148	-0.013	-0.040	0.103	0.205	-0.106	-0.040	0.011	0.122	0.039	-0.005	-0.052	-0.039	-0.074	-0.038	-0.038	1

Supplementary Table 5: methodological steps for the building of the Governance and Social Vulnerability dimensions

**Dimension**	**Additional data selection step**	**Missing values and extreme values**	**Normalisation and inversion**	**Aggregation and weighing**
Governance	2018 estimations of the "percentile rank among all countries" for each of the six Worldwide Governance Indicators	Missing values were rare (11 points). For cases with missing values, we assigned a value equal to the average of WGI scores of the whole sample.	Percentile values were converted to fractions and reversed so that a high score corresponds to a high risk.	The total Governance score is the average of these six indicator scores.
Social Vulnerability	No additional step	Total Dependency Ratio (TDR): i) cases with missing values were assigned the average country value, calculated from the individual values of the mining projects located in that country. ii) Ratio was capped to 200 in order to reduce the incidence of rare extreme values (17 points).	The three variables were normalised using the the formula: Xnorm = (X – Xmin) / (Xmax – Xmin), Xmax (Xmin) being the value of the country scoring the highest (lowest) according to the variable.<br>Human Development Index (HDI): normalised value was reversed so that a high score corresponds to a high risk.	Social Vulnerability score calculated by aggregating the normalised values of the HDI, Gini coefficient and TDR with a weight of 0.6, 0.2 and 0.2 respectively. The difference in weighing reflects the fact that the three variables are different in nature. The HDI comprises three dimensions that use their own specific measures, while the GINI coefficient and the TDR are individual measures. The GINI coefficient is a statistical measure of wealth distribution and the TDR is a percentage. Weights were adjusted to 0.75 for the HDI and 0.25 for the TDR when the Gini coefficient value was missing (150 points). When both HDI and Gini values were missing (33 points), we sought alternative values calculated from the average score of neighbouring countries.

Supplementary Table 6: methodological steps for the building of the Land Uses and Conservation dimensions

**Dimension**	**Additional data selection step**	**Missing values and extreme values**	**Normalisation and inversion**	**Aggregation and weighing**
Communities	The Communities dimension is made of three variables: i) the Global Human Settlements Layer (GHSL) population density value of the 1 km^2 cell in which the mining project point falls; ii) The sum of GHSL population densities of cells falling within a 100 km buffer zone around the point; iii) the Indigenous Peoples Land polygons.	No missing or extreme value issue	The normalisation process accounts for the fact that levels of vulnerability of local communities are not directly proportional to the number of human lives at stake. Any non-zero population density within 1km of the mine location was interpreted as a maximum risk score of 1.<br>For variable (ii), i.e. population density within a 100 km buffer, the score was set equal to log(1+x) / log(1+xmax).	The base communities score is made of the average of the two population density variables. Mining properties falling into a polygon of the Indigenous Peoples Land dataset had their Communities score increased by 0.2, to account for the added level of vulnerability in indigenous communities.
Land Uses	No additional step	For Pasture Land and Cropland variables, missing values were rare and generally correspond to remote areas. They were therefore assigned a value of zero.	The three variables were divided by their maximum value to obtain normalised values.	Because Pasture Land and Cropland datasets are issued by the same source and use the same cell resolution, their summation is also a percentage of occupied land. The percentage not occupied by either pastures or crops can be occupied by forests. The three variables were therefore aggregated using the formula: $\text{Crops} + \text{Pastures} + (1 - \text{Crops} - \text{Pastures}) * \text{Forests}$. This prevents overlap between data from different sources.

Supplementary Table 7: methodological steps for the building of the Conservation, Water and Waste dimensions

Dimension	Additional data selection step	Missing values and extreme values	Normalisation and inversion	Aggregation and weighing
Conser-vation	The Conservation category is built on the distance from a mining project to key or threatened biodiversity polygons, and values of total species richness in the location of the mining project. Distance to the nearest biodiversity polygon (either from the Key Biodiversity Area dataset or the Threatened Biodiversity Hotspots dataset) was calculated with the NEAR function of ArcGIS 10 Spatial Analyst. The total species richness is made of the sum of all species richness rasters.	No missing or extreme value issue	Distances were rescaled by their rank order such that mining projects falling within a polygon get the highest risk value (1) and that mining projects furthest away from any polygon get the minimum risk value (0).<br>Total species richness values are normalised by the maximum value across the sample.	The Conservation score is the average of the normalised distance and richness scores.
Water	No additional step	Missing data for the Baseline Water Stress (38 points) were given a value of 5, i.e. maximum risk, on the basis that the cases were located in either remote islands or in Greenland, which are locations with specific water challenges. Cases with missing value for the Inter-annual Variability were given a Water score solely based on their Baseline Water Stress.	The Baseline Water Stress and the Inter-annual Variability are already expressed as a risk scale (from 0 for the lowest risk to 5 for extremely high risk). Values were divided by 5 for normalisation.	The Water score is the average of the two indicator scores.
Waste	The precipitation variable was built by taking the maximum value out of the 12 monthly values recorded by the WorldClim dataset. This step accounts for the influence of heavy rains on mine waste containment failures.	No missing or extreme value issue	For each of the six indicators we took the percentile rank to generate an even distribution of scores between 0 and 1.	The Waste score is the average of the five indicator scores. This step accounts for the cumulative effect the five factors can have on waste containment failures.

Supplementary table 8: Selected mining projects and associated contained resources (source: S&P database)

	Number of mining projects			Amount of contained resources			
	Total	Operating	Pre-production	Total	Operating	Pre-production	Unit
**Silver**	1702	441	1261	6.31E+10	3.23E+10	3.08E+10	ounces
**Bauxite**	102	50	52	1.31E+10	7.51E+09	5.61E+09	tonnes
**Cobalt**	280	57	223	2.30E+07	1.27E+07	1.02E+07	tonnes
**Copper**	1580	455	1125	2.58E+09	1.68E+09	9.00E+08	tonnes
**Iron**	618	198	420	2.49E+11	1.30E+11	1.19E+11	tonnes
**Lanthanides**	103	11	92	2.08E+08	6.72E+07	1.41E+08	tonnes
**Lead**	694	219	475	2.55E+08	1.35E+08	1.20E+08	tonnes
**Lithium**	113	26	87	1.81E+08	9.59E+07	8.46E+07	tonnes
**Manganese**	64	30	34	1.40E+09	9.74E+08	4.26E+08	tonnes
**Molybdenum**	345	84	261	5.76E+07	2.47E+07	3.29E+07	tonnes
**Nickel**	449	101	348	3.31E+08	1.33E+08	1.99E+08	tonnes
**Scandium**	8	0	8	7.80E+04	0.00E+00	7.80E+04	tonnes
**Tin**	60	14	46	5.76E+06	2.46E+06	3.30E+06	tonnes
**Yttrium**	17	1	16	1.37E+06	3.41E+04	1.33E+06	tonnes
**Zinc**	950	266	684	7.34E+08	4.08E+08	3.26E+08	tonnes
**Gold**	3955	1029	2926	6.36E+09	3.09E+09	3.27E+09	ounces
**Platinum**	171	50	121	2.29E+09	1.68E+09	6.11E+08	ounces
**Heavy Mineral Sands**	53	14	39	2.00E+09	4.73E+08	1.53E+09	tonnes
**Tungsten**	106	37	69	1.07E+07	4.75E+06	5.96E+06	tonnes
**U3O8**	372	62	310	2.57E+10	1.01E+10	1.56E+10	pounds
**Total sample**	6888	1884	5004	N.A.	N.A.	N.A.	N.A.

Supplementary Table 9: Top 15 countries according to sum of total ESG score for selected metal groups.

Global Rank	All 20 ETMs	Figure 2c ETMs	Cobalt, rare earths, lithium - metals with highest relative demand increase	Rare earths, iron and lithium - metals with a comparatively low-risk profile	Iron, copper and nickel - metals with highest cumulative mined ore tonnage	Platinum, cobalt and silver - metals with a comparatively high-risk profile	Copper, aluminium and nickel - metals with a comparatively medium-risk profile
1	Australia	Australia	Australia	Australia	Australia	Mexico	Australia
2	United States	United States	United States	China	China	United States	China
3	China	China	Canada	Brazil	Canada	Australia	Brazil
4	Canada	Canada	Congo (DRC)	Canada	United States	Canada	Canada
5	Mexico	Mexico	China	United States	Russia	Peru	South Africa
6	Russia	Russia	Argentina	Russia	Peru	China	Russia
7	Peru	Peru	Philippines	South Africa	Mexico	South Africa	Philippines
8	South Africa	South Africa	Finland	Chile	Chile	Russia	Indonesia
9	Brazil	Brazil	Brazil	Philippines	Brazil	Argentina	India
10	Chile	Chile	Zambia	Argentina	Philippines	Indonesia	United States
11	Philippines	Philippines	Russia	India	South Africa	Congo (DRC)	Guinea
12	Kazakhstan	Indonesia	Namibia	Mexico	Congo (DRC)	Chile	Chile
13	Indonesia	Argentina	Cuba	Sweden	Kazakhstan	Turkey	Finland
14	Argentina	Zimbabwe	Tanzania	Namibia	Indonesia	Philippines	Sweden
15	Zimbabwe	Congo (DRC)	Chile	Nigeria	Zambia	Ecuador	Cameroon

Supplementary Table 10: Top 10 hot spot countries according to sum of total ESG score and average total ESG scores.

Country	Mining projects per km2	Mining projects	Ag (%)	Bx (%)	Co (%)	Cu (%)	Fe (%)	La (%)	Pb (%)	Li (%)	Mn (%)	Mo (%)	Ni (%)	Zn (%)	Pt (%)	Sum of total scores	Average total score
China	0.61	575	0	7.63	1.18	3.41	0	34.3	10.7	6.55	1.69	16.8	2.72	9.42	0.18	2100	3.65
Mexico	1.38	270	0	0	1.05	2.56	2.00	0	6.32	1.97	1.12	3.34	0	7.33	0	1020	3.78
Peru	1.63	211	0	0	0	8.15	10.4	0	5.38	1.14	0.24	7.35	0	8.10	0	754	3.57
South Africa	1.57	191	1.05	0	0.24	0.30	0	0.09	1.74	0	53.3	0	4.14	2.54	84.43	684	3.58
Philippines	3.41	101	0.26	3.79	4.44	1.20	1.58	0	0.02	0	0	0.36	10.2	0.01	0	374	3.70
Kazakhstan	0.39	106	6.50	2.63	0	2.51	0	0	3.05	0.15	4.40	5.70	0.33	2.79	0	370	3.49
Indonesia	0.44	83	6.64	7.89	1.16	2.44	3.65	0	1.40	0	0.06	0.11	16.8	0.76	0	291	3.51
Zimbabwe	1.43	56	0	0	0	0.05	0	0	0	0.71	0	0	0.46	0.07	4.57	233	4.15
Congo DRC	0.23	54	0.08	0	49.2	3.69	0	0	0.05	3.67	0	0	0	0.64	0	216	4.00
India	0.15	47	6.22	11.31	0.04	0.05	0	1.73	3.78	0	0	0	0.09	3.80	0.03	167	3.55

Supplementary Table 11: Top 10 cold spot countries according to number of mining projects, mining project concentration and average total ESG scores

Country	Mining projects per km2	Mining projects	Ag (%)	Bx (%)	Co (%)	Cu (%)	Fe (%)	La (%)	Pb (%)	Li (%)	Mn (%)	Mo (%)	Ni (%)	Zn (%)	Pt (%)	Sum of total scores	Average total score
Australia	1070	1.39	0	0	10.4	5.17	0	3.34	24.2	8.14	11.3	2.96	12.8	15.1	0.16	2760	2.58
Canada	1068	1.08	0	0	4.84	4.07	0	19.3	7.58	4.03	2.93	8.71	6.34	10.2	0.73	1657	1.55
United States	859	0.92	0	0	2.20	8.42	7.87	3.23	6.89	12.4	1.03	24.9	2.76	5.95	4.19	2336	2.72
Russia	319	0.19	0	0	1.11	4.82	0	12.3	5.04	0	0.02	2.61	10.8	10.0	5.32	883	2.77
Chile	133	1.76	8.96	0	0.64	34.9	0	0	0.04	12.6	0.16	14.8	0	0.13	0	390	2.93
Argentina	84	0.30	0	0	0.01	2.54	0.79	0.06	0.91	20.4	0	2.57	0.01	0.18	0.00	250	2.98
Sweden	62	1.38	4.74	0	0.02	0.39	9.37	0.12	1.32	0	0	2.17	1.27	1.57	0.00	119	1.92
Finland	51	1.52	0.20	0	0.97	0.12	0.50	0.01	0.01	0.07	0	0	0.48	0.05	0.23	106	2.07
Mongolia	43	0.27	4.69	0	0	2.36	2.23	2.09	0.48	0	0	1.93	0	0.36	0	137	3.18
Spain	31	0.61	2.89	0	0.03	0.25	0.17	0	1.34	0.46	0.02	0	0	1.31	0	93	2.99

Supplementary Figures

Waste	Water	Conservation	Communities	Land Uses	Social Vulnerability	Governance	Environment	Social		Total score
0.27	0.6	0.67	0.58	0.55	0.47	0.45	0.51	0.68	Platinum	3.6
0.46	0.31	0.47	0.56	0.63	0.49	0.66	0.41	0.78	Cobalt	3.6
0.33	0.68	0.3	0.52	0.7	0.39	0.44	0.44	0.68	Manganese	3.4
0.45	0.43	0.59	0.47	0.52	0.37	0.39	0.49	0.59	Heavy Mineral Sands	3.2
0.44	0.19	0.6	0.61	0.5	0.32	0.48	0.41	0.64	Tin	3.1
0.44	0.48	0.5	0.5	0.48	0.27	0.43	0.47	0.56	Silver	3.1
0.43	0.4	0.45	0.63	0.56	0.22	0.42	0.43	0.61	Tungsten	3.1
0.48	0.58	0.5	0.52	0.34	0.29	0.38	0.52	0.51	Copper	3.1
0.42	0.46	0.49	0.58	0.48	0.24	0.39	0.46	0.56	Lead	3.1
0.48	0.4	0.5	0.45	0.42	0.33	0.47	0.46	0.56	Nickel	3.0
0.41	0.45	0.49	0.56	0.44	0.27	0.43	0.45	0.57	Zinc	3.0
0.41	0.14	0.51	0.38	0.55	0.45	0.59	0.35	0.66	Aluminium	3.0
0.43	0.4	0.47	0.47	0.44	0.31	0.43	0.43	0.55	Gold	2.9
0.45	0.54	0.45	0.51	0.4	0.23	0.34	0.48	0.49	Molybdenum	2.9
0.41	0.46	0.43	0.47	0.48	0.25	0.41	0.43	0.54	Rare Earths	2.9
0.42	0.69	0.43	0.25	0.5	0.23	0.38	0.51	0.45	Yttrium	2.9
0.45	0.35	0.49	0.39	0.46	0.28	0.4	0.43	0.51	Iron	2.8
0.34	0.6	0.49	0.45	0.2	0.3	0.44	0.48	0.46	Lithium	2.8
0.3	0.56	0.39	0.4	0.4	0.23	0.27	0.42	0.43	U3O8	2.5
0.32	0.38	0.35	0.31	0.57	0.16	0.1	0.35	0.38	Scandium	2.2

Environment — Social

Supplementary Figure 1: Environmental Social and Governance analysis results for the 20 commodities analysed

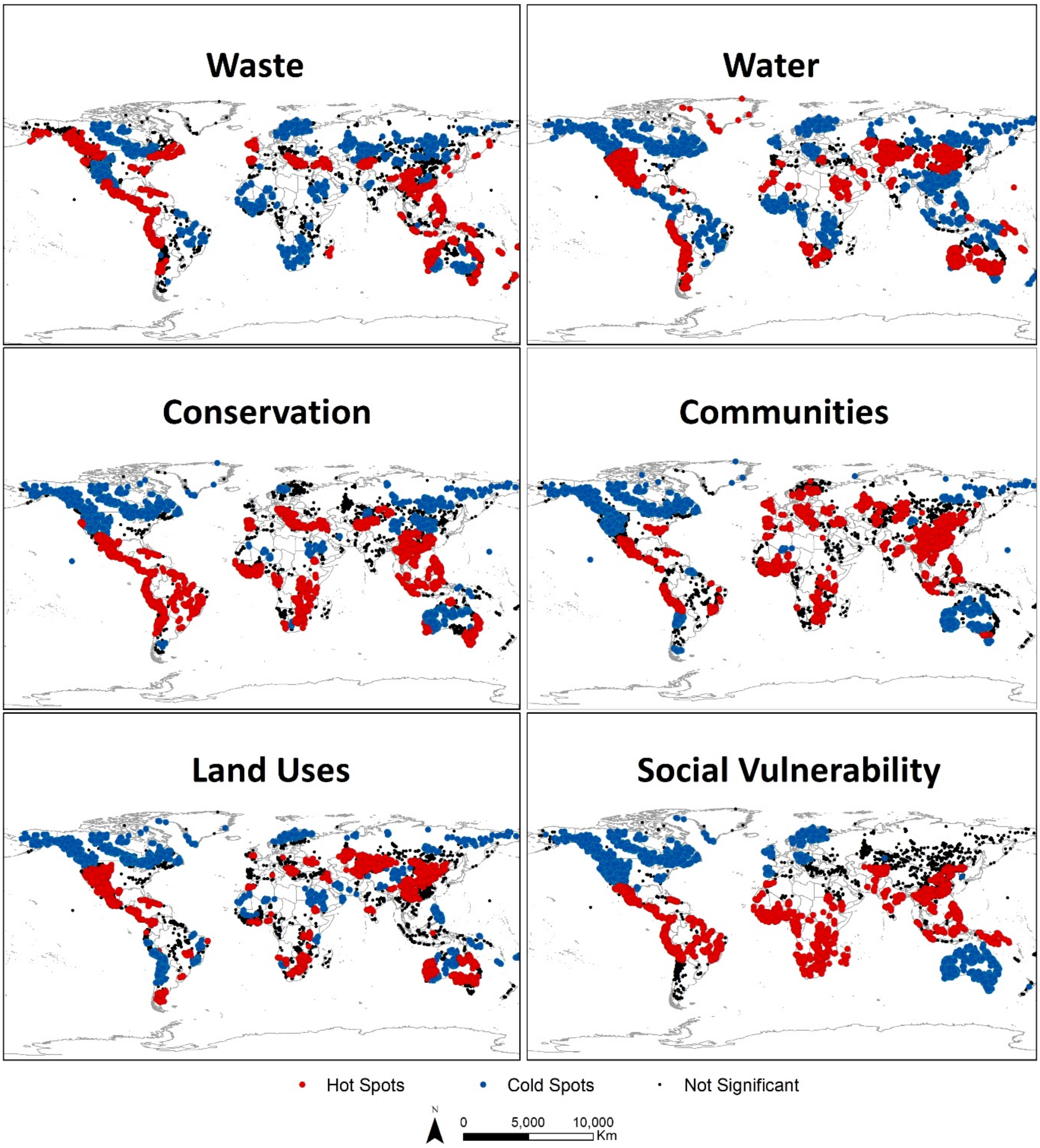

Supplementary Figure 2: hot and cold spots distribution for each risk dimension, all metals combined

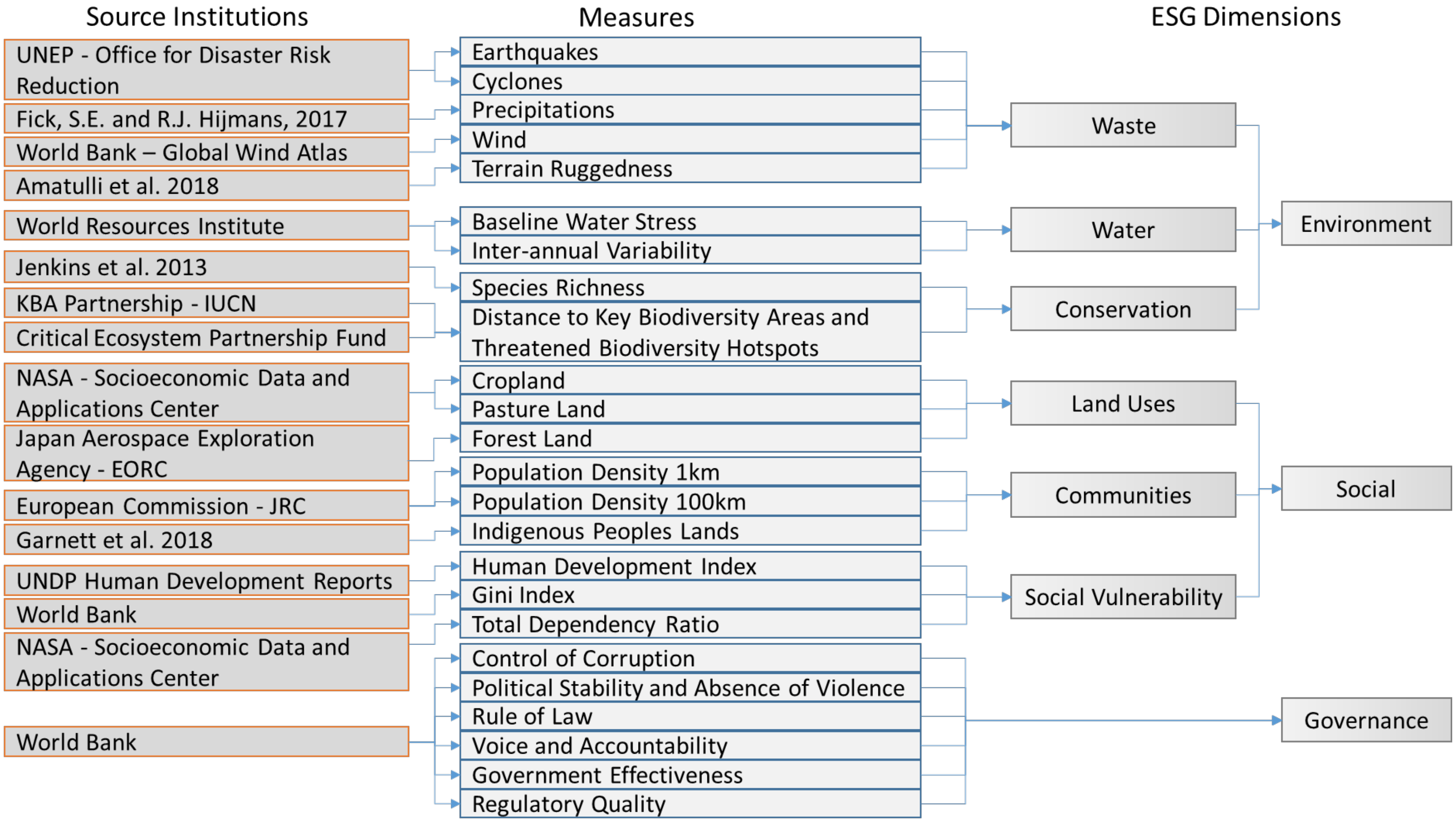

Supplementary Figure 3: ESG framework structure, indicators and source institution

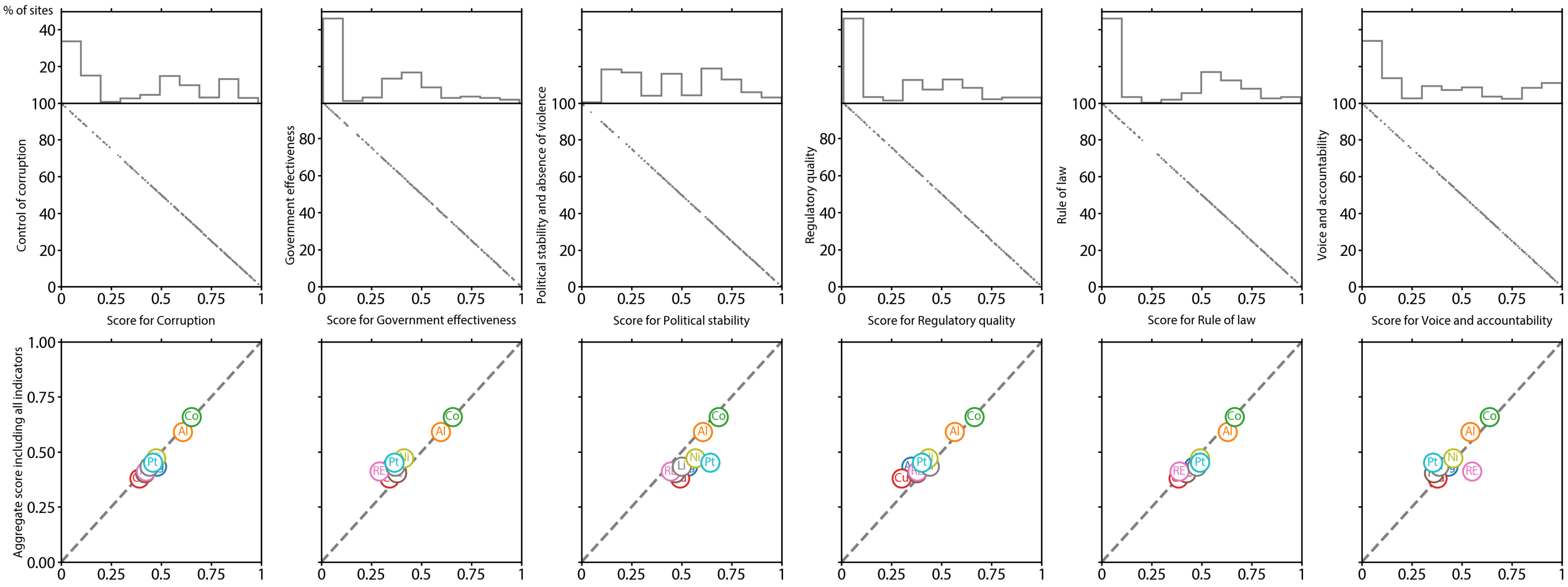

Supplementary Figure 4: Distribution of individual governance indicators (top row) and contribution of each indicator to the overall governance dimension (bottom row)

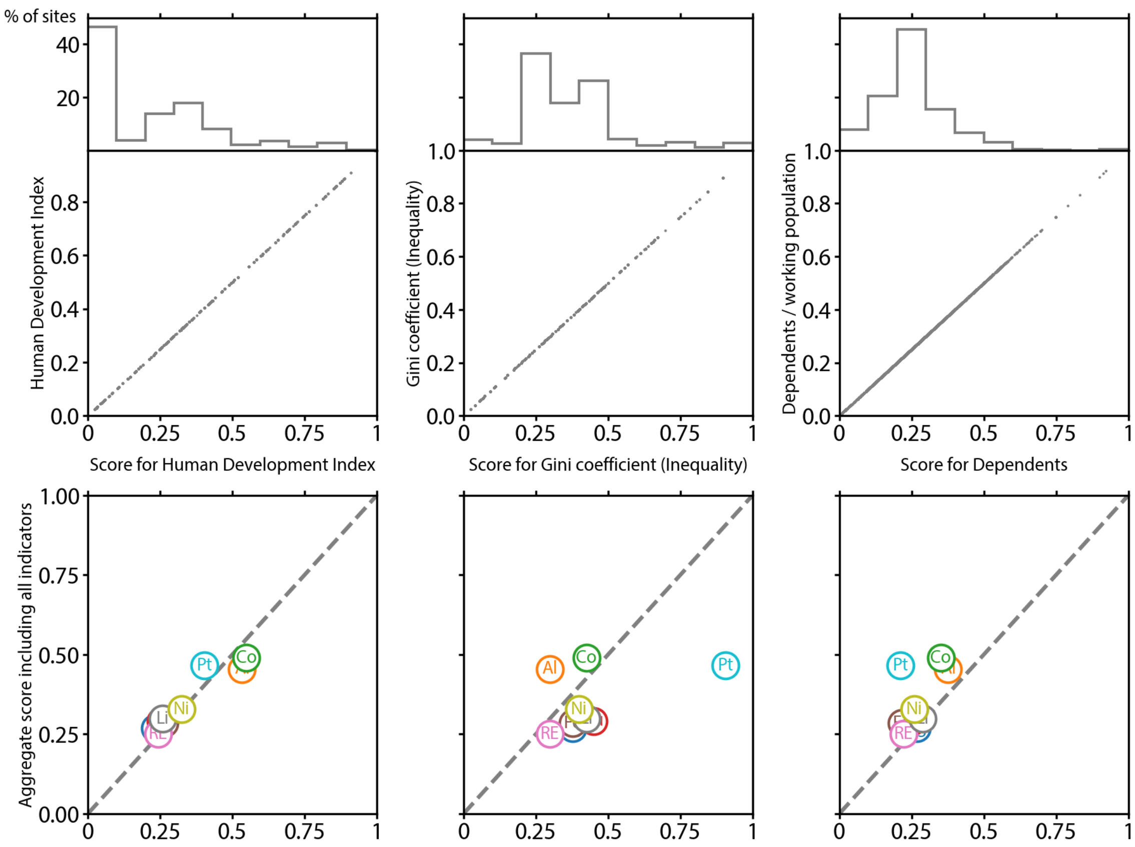

Supplementary Figure 5: Distribution of individual Social Vulnerability indicators (top row) and contribution of each indicator to the overall Social Vulnerability dimension (bottom row)

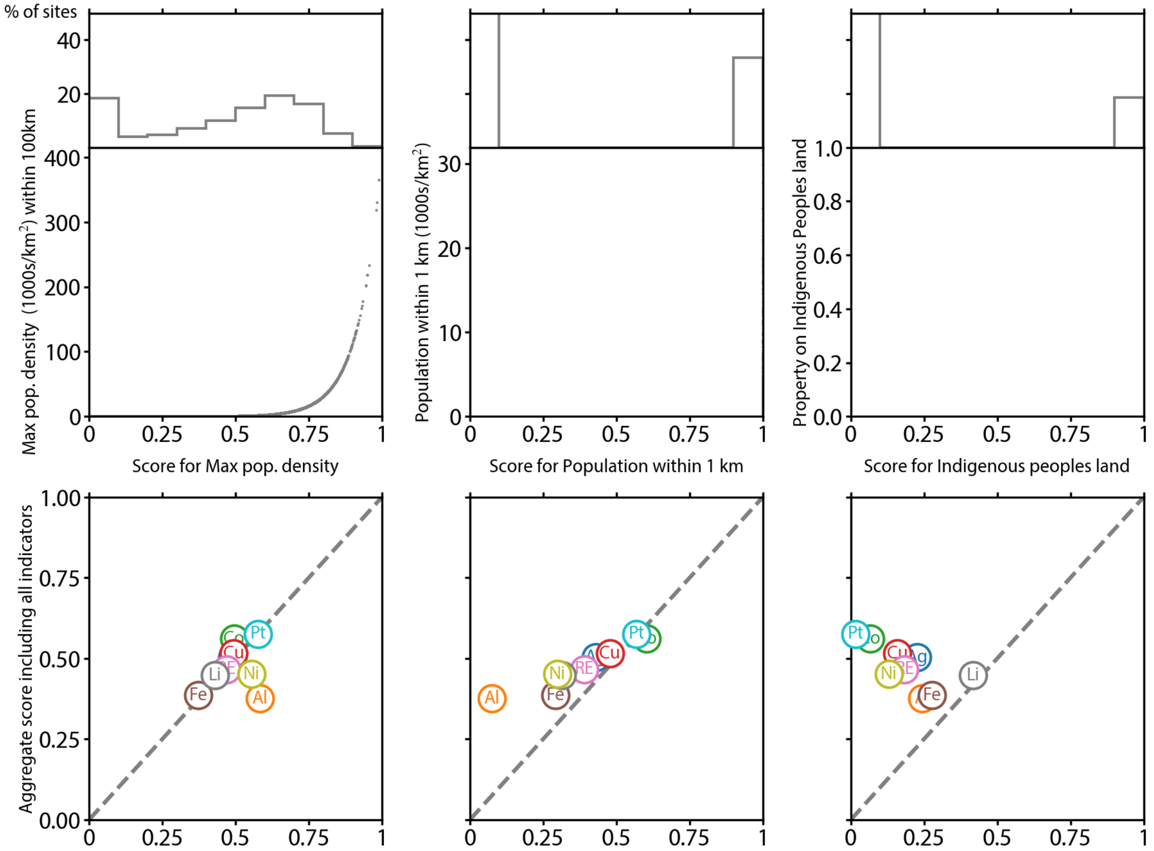

Supplementary Figure 6: Distribution of individual communities indicators (top row) and contribution of each indicator to the overall communities dimension (bottom row)

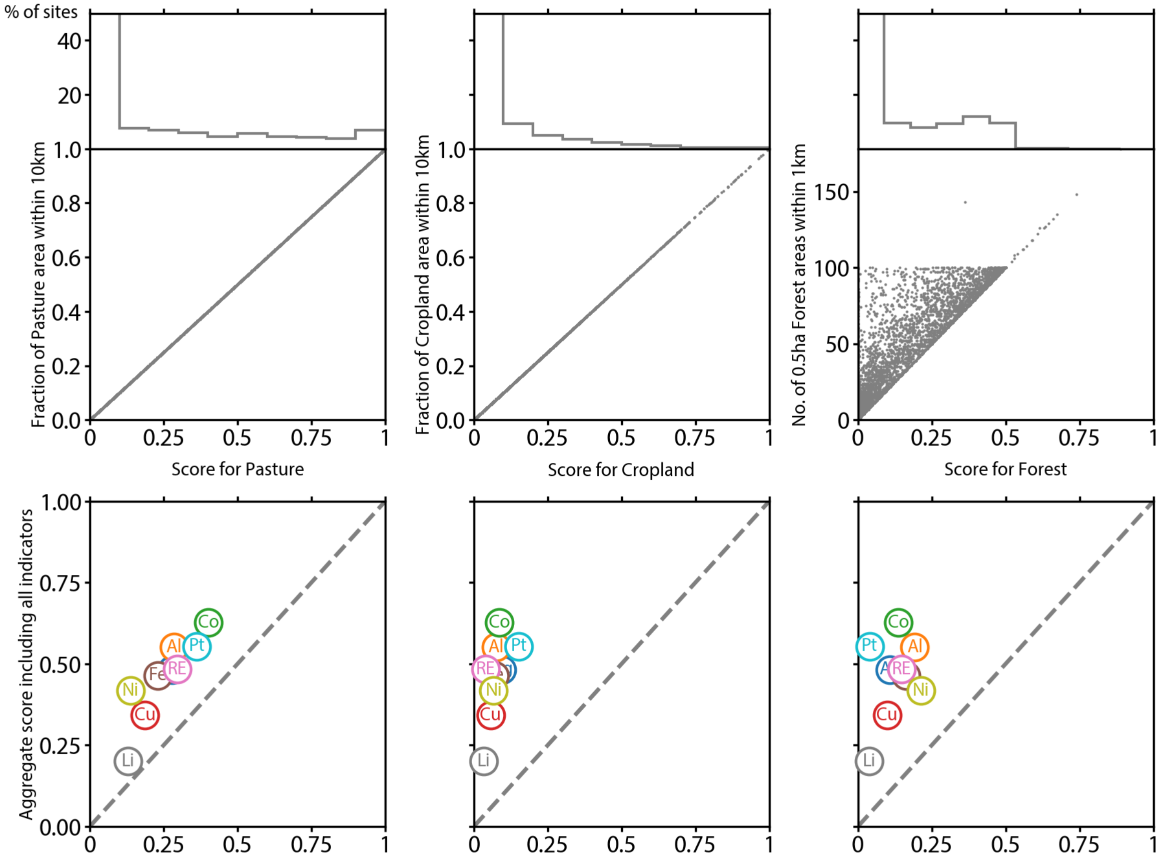

Supplementary Figure 7: Distribution of individual land use indicators (top row) and contribution of each indicator to the overall land use dimension (bottom row)

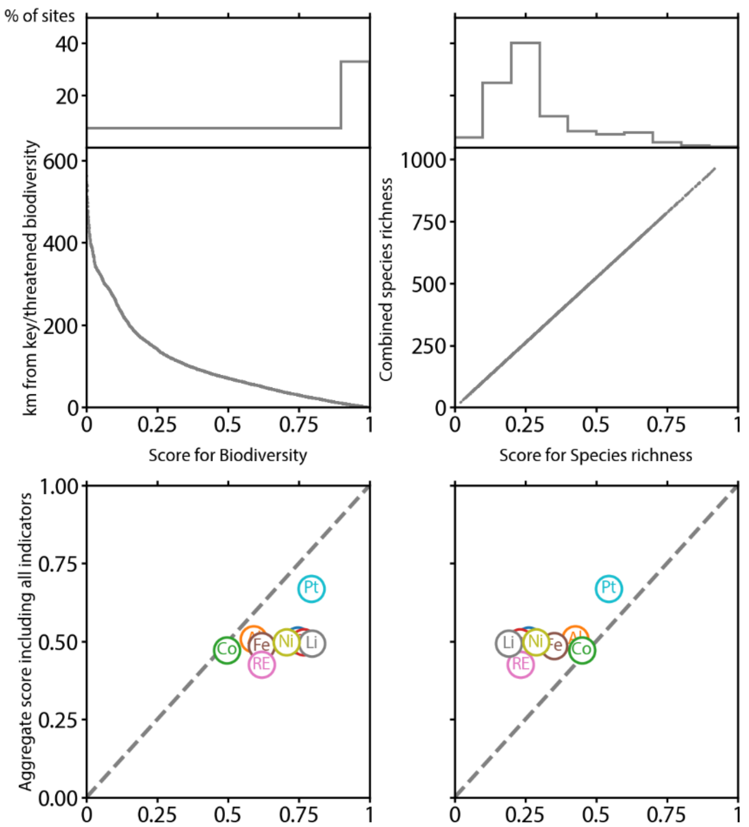

Supplementary Figure 8: Distribution of individual conservation indicators (top row) and contribution of each indicator to the overall conservation dimension (bottom row)

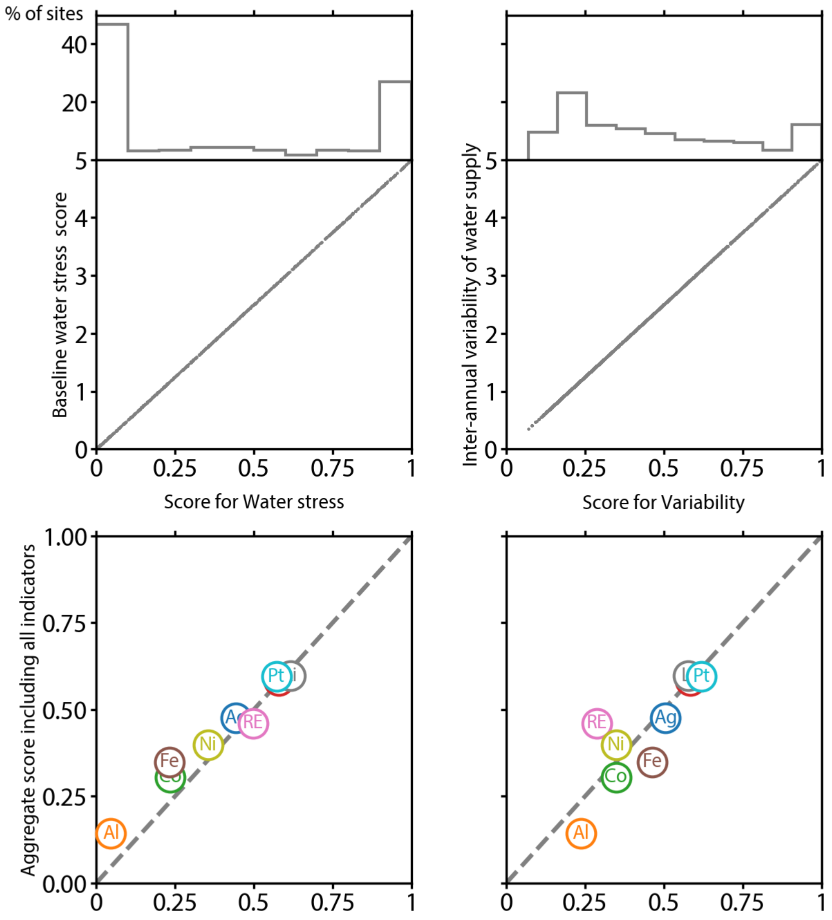

Supplementary Figure 9: Distribution of individual water indicators (top row) and contribution of each indicator to the overall water dimension (bottom row)

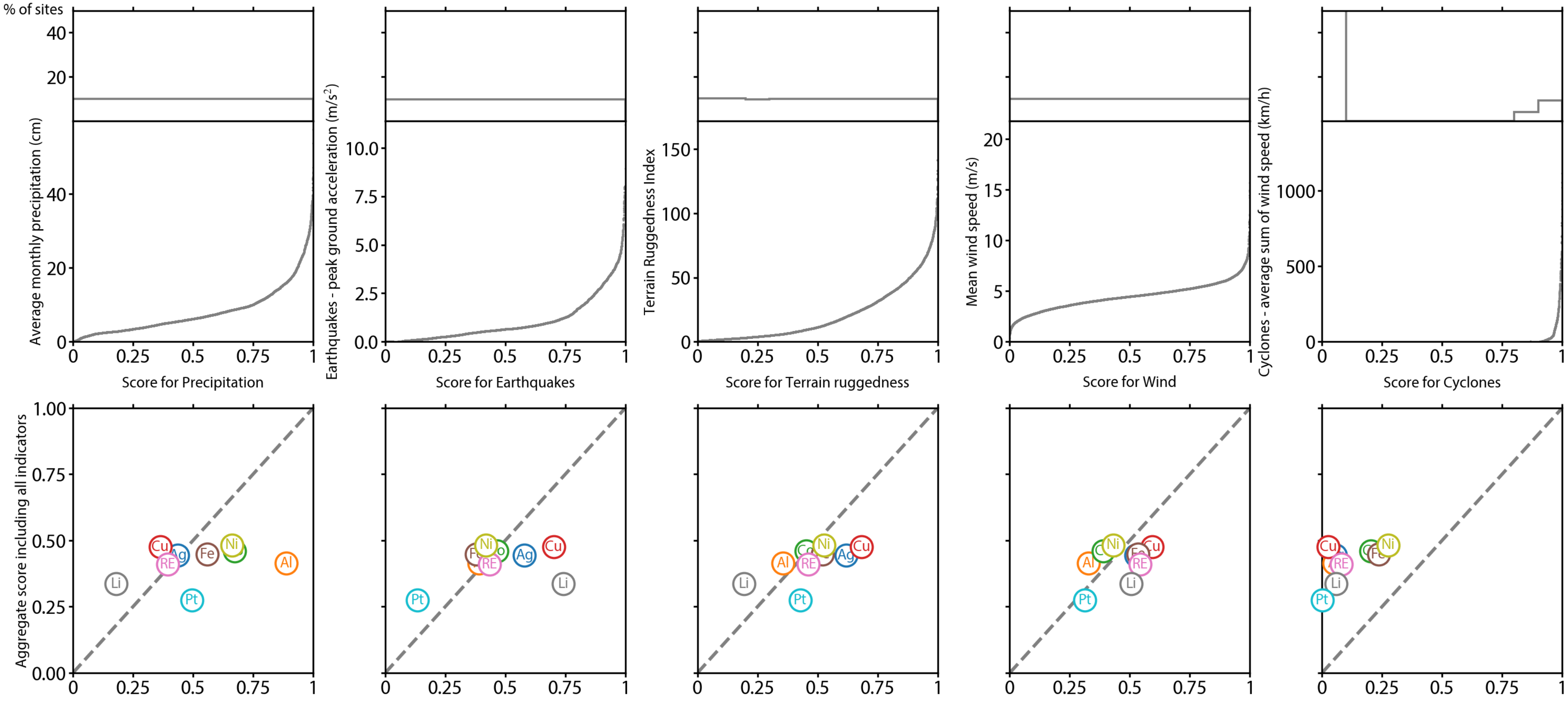

Supplementary Figure 10: Distribution of individual waste indicators (top row) and contribution of each indicator to the overall waste dimension (bottom row)

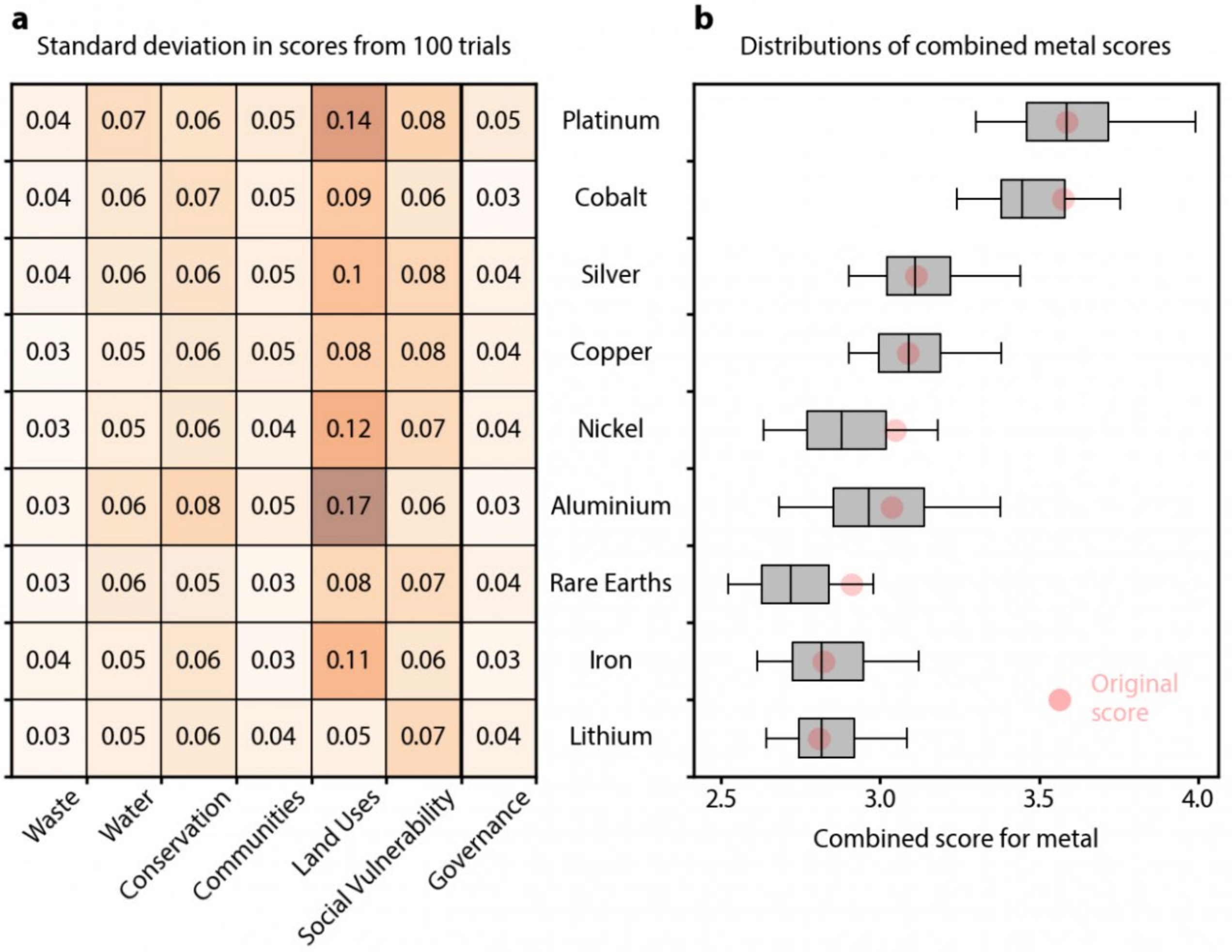

Supplementary Figure 11: Sensitivity analysis for the ESG risk matrix (n = 6888 mining projects), testing the stability of each risk dimension (a) and the total ESG score (b). The test is to deliberately skew each risk dimension by raising them to a random power between 0.5 and 2. Running this for 100 trials, using different sets of random numbers for each trial, we get to see how much the aggregate scores depend on the precise scaling of the individual indicators. Boxes in panel b show the mean (vertical bar) and interquartile range of literature estimates (n=17), and error bars show the 5th and 95th percentiles.

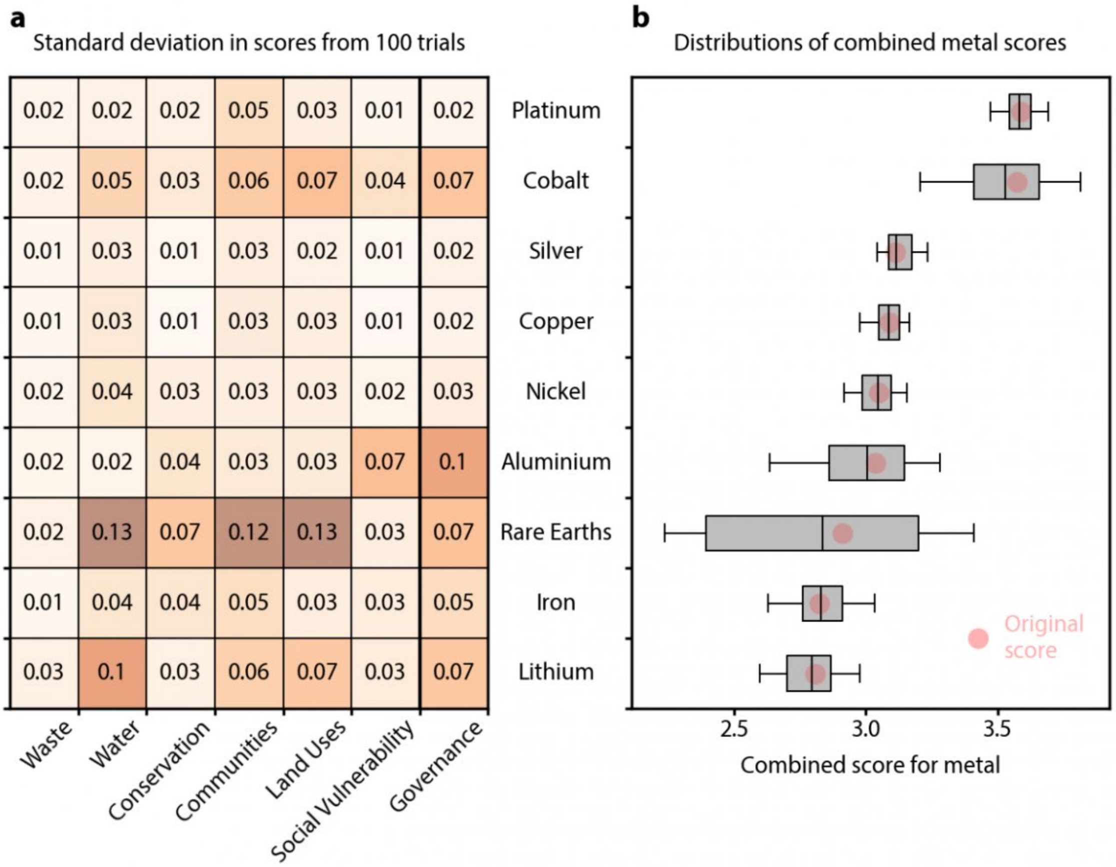

Supplementary Figure 12: Completeness test for each risk dimension (a) and the total ESG score (b). The completeness test re-runs the analysis 100 times, each time with a different - randomly selected - subset of the data. Subsets used represent 90% of the complete set. Each trial is with a different set of 6199 mining projects taken the full set of 6888 mining projects. Boxes in panel b show the mean (vertical bar) and interquartile range of literature estimates (n=17), and error bars show the 5th and 95th percentiles.

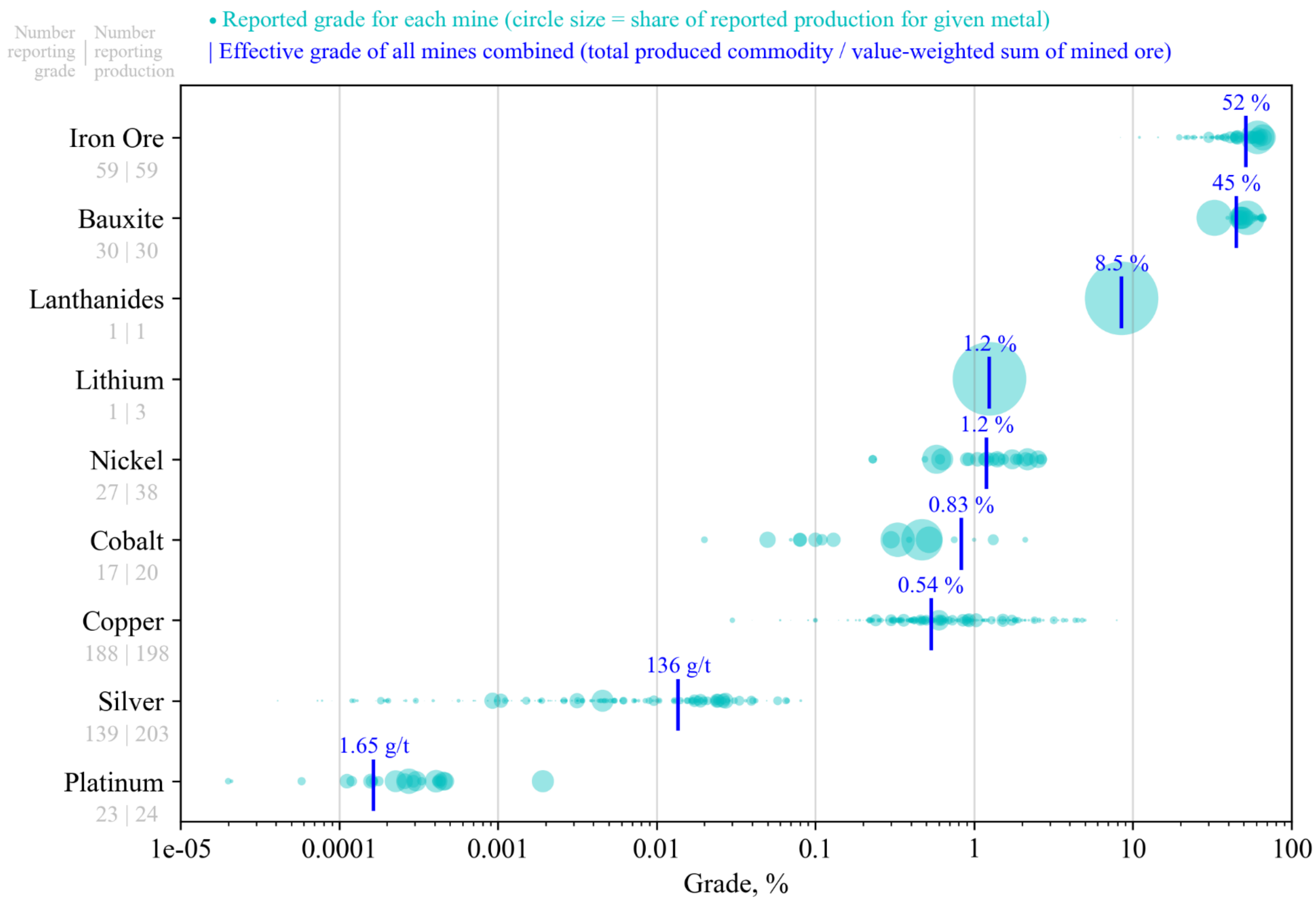

Supplementary Figure 13: Grades and production values reported in the S&P database for the 9 commodities in Figure 1

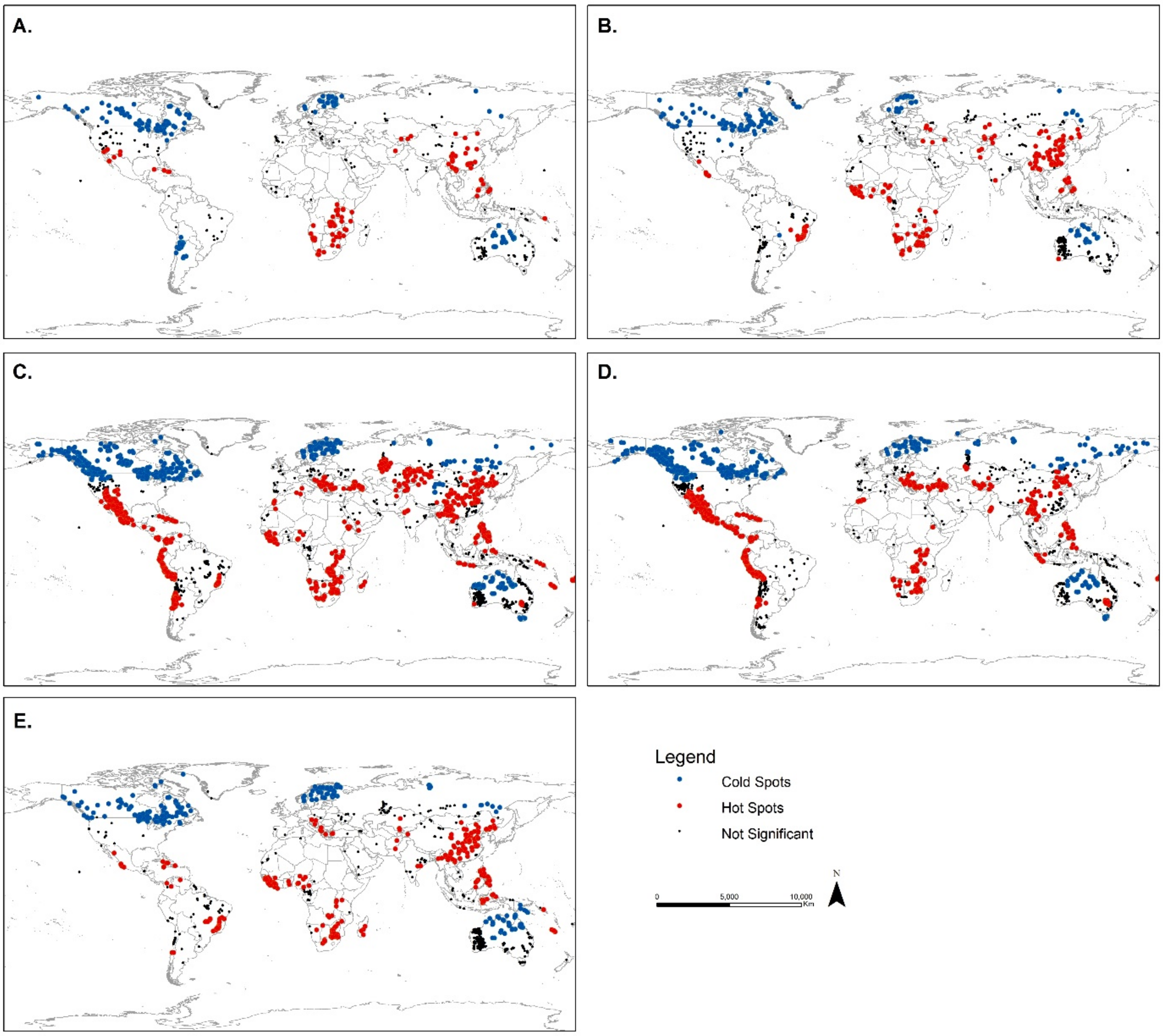

Supplementary Figure 14: hot and cold spots distribution for selected metal groups. A. metals with highest relative demand increase, cobalt, rare earths and lithium; B. metals with a comparatively low-risk profile, rare earths, iron and lithium; C. metals with highest cumulative mined ore tonnage, iron, copper and nickel; D. metals with a comparatively high-risk profile, platinum, cobalt and silver; E. metals with a comparatively medium-risk profile, copper, aluminium and nickel.

Supplementary Note 1

On the Waste dimension

The relationship between waste containment failure events and external factors is complex. Analyses of past catastrophic tailings dam failures often identify several underlying causes (Rico et al. 2008), some of which are external (heavy rains, seismic events), and some internal (management decisions, human error). In building this category, we acknowledge the diversity of potentially contributing factors and a cumulative effect. The five indicators we use have been acknowledged as contributing external factors in the literature. Their specific connection to containment issues are listed below:

1) Seismicity: catastrophic tailings dam failures (LPSDP 2016, WISE 2020)
2) Cyclone intensity: catastrophic tailings dam failures and airborne pollution (Azam & Li 2010, Rico et al. 2008)
3) Wind speed: airborne pollution (Balabanova et al. 2012)
4) Maximum precipitations: catastrophic tailings dam failures and acid mine drainage (WISE 2020, Rico et al. 2008)
5) Terrain ruggedness: catastrophic tailings dam failures and acid mine drainage (Rico et al. 2008, LPSDP 2016)

These five indicators, however, only represent an approximation of a complex system. In particular, these indicators do not account for faults in the design and control of tailings dams (i.e. human responsibility) which are the most common sources of tailings dam failures (LPSDP 2016).

Supplementary References

1. Davidson, V., *Copper market outlook: Transitioning to deficits*, in *Copper to the World Conference*. 2017, Government of South Australia. Department for Energy and Mining: Adelaide Convention Centre.
2. de Koning, A., et al., *Metal supply constraints for a low-carbon economy?* Resources, Conservation and Recycling, 2018. **129**: p. 202-208.
3. Deetman, S., et al., *Scenarios for demand growth of metals in electricity generation technologies, cars, and electronic appliances.* Environmental science & technology, 2018. **52**(8): p. 4950-4959.
4. Elshkaki, A. and T. Graedel, *Solar cell metals and their hosts: a tale of oversupply and undersupply.* Applied energy, 2015. **158**: p. 167-177.
5. Giurco, D., et al., *Requirements for Minerals and Metals for 100% Renewable Scenarios*, in *Achieving the Paris Climate Agreement Goals: Global and Regional 100% Renewable Energy Scenarios with Non-energy GHG Pathways for +1.5°C and +2°C*, S. Teske, Editor. 2019, Springer International Publishing: Cham. p. 437-457.
6. Harvey, L.D., *Resource implications of alternative strategies for achieving zero greenhouse gas emissions from light-duty vehicles by 2060.* Applied energy, 2018. **212**: p. 663-679.
7. Hertwich, E.G., et al., *Integrated life-cycle assessment of electricity-supply scenarios confirms global environmental benefit of low-carbon technologies.* Proceedings of the National Academy of Sciences, 2015. **112**(20): p. 6277-6282.
8. Hund, K.L., et al., *Minerals for Climate Action: The Mineral Intensity of the Clean Energy Transition.* 2020.
9. Li, W. and T. Adachi, *Evaluation of long-term silver supply shortage for c-Si PV under different technological scenarios.* Natural Resource Modeling, 2019. **32**(1): p. e12176.
10. Månberger, A. and B. Stenqvist, *Global metal flows in the renewable energy transition: Exploring the effects of substitutes, technological mix and development.* Energy Policy, 2018. **119**: p. 226-241.
11. Pehlken, A., S. Albach, and T. Vogt, *Is there a resource constraint related to lithium ion batteries in cars?* The International Journal of Life Cycle Assessment, 2017. **22**(1): p. 40-53.
12. Rasmussen, K.D., et al., *Platinum Demand and Potential Bottlenecks in the Global Green Transition: A Dynamic Material Flow Analysis.* Environmental Science & Technology, 2019. **53**(19): p. 11541-11551.
13. Valero, A., et al., *Material bottlenecks in the future development of green technologies.* Renewable and Sustainable Energy Reviews, 2018. **93**: p. 178-200.
14. Watari, T., K. Nansai, and K. Nakajima, *Review of critical metal dynamics to 2050 for 48 elements.* Resources, Conservation and Recycling, 2020. **155**: p. 104669.
15. Watari, T., et al., *Total material requirement for the global energy transition to 2050: A focus on transport and electricity.* Resources, Conservation and Recycling, 2019. **148**: p. 91-103.
16. Watari, T., et al., *Analysis of potential for critical metal resource constraints in the international energy agency's long-term low-carbon energy scenarios.* Minerals, 2018. **8**(4): p. 156.
17. Ziemann, S., et al., *Modeling the potential impact of lithium recycling from EV batteries on lithium demand: a dynamic MFA approach.* Resources, Conservation and Recycling, 2018. **133**: p. 76-85.
18. USGS, *Mineral Commodity Summaries 2020*. 2020, U.S. Geological Survey: Reston, VA, USA. p. 200.
19. S&P, *S&P Global Market Intelligence*. 2019, Thomson Reuters: New York.
20. Giardini, D., et al., *The GSHAP Global Seismic Hazard Map*, W. Lee, et al., Editors. 2003, International Handbook of Earthquake & Engineering Seismology, Academic Press: Amsterdam.

21. UNEP/GRID-Europe, *Tropical cyclones average sum of windspeed 1970-2009*. 2014.
22. Global Wind Atlas, *World - Wind Speed And Wind Power Potential Maps*. 2017, World Bank.
23. Amatulli, G., et al., *A suite of global, cross-scale topographic variables for environmental and biodiversity modeling.* Scientific data, 2018. **5**: p. 180040.
24. Gassert, F., et al., *Aqueduct Metadata Document - Aqueduct Global Maps 2.0*. 2013, World Resources Institute: Washington DC, United States.
25. Jenkins, C.N., S.L. Pimm, and L.N. Joppa, *Global patterns of terrestrial vertebrate diversity and conservation.* Proceedings of the National Academy of Sciences, 2013. **110**(28): p. E2602-E2610.
26. BirdLife International, *Digital boundaries of Important Bird and Biodiversity Areas from the World Database of Key Biodiversity Areas*. 2019.
27. CEPF, *Biodiversity Hotspots Defined*. 2020, Critical Ecosystem Partnership Fund, Conservation International.
28. Ramankutty, N., et al., *Global Agricultural Lands: Pastures, 2000*. 2010, NASA Socioeconomic Data and Applications Center (SEDAC): Palisades, NY.
29. Ramankutty, N., et al., *Global Agricultural Lands: Croplands, 2000*. 2010, NASA Socioeconomic Data and Applications Center (SEDAC): Palisades, NY.
30. Shimada, M., et al., *New global forest/non-forest maps from ALOS PALSAR data (2007–2010).* 2014. **155**: p. 13-31.
31. Florczyk, A.J., et al., *GHSL Data Package 2019*, in *JRC Technical Reports*. 2019, Publications Office of the European Union: Luxembourg.
32. Garnett, S.T., et al., *A spatial overview of the global importance of Indigenous lands for conservation.* Nature Sustainability, 2018. **1**(7): p. 369-374.
33. UNDP, *Human Development Index*, in *Human Development Reports* 2018, United Nations Development Programme.
34. World Bank, *Gini Index - Years 2008 - 2017*. 2018.
35. Center for International Earth Science Information Network - CIESIN - Columbia University, *Gridded Population of the World, Version 4 (GPWv4): Basic Demographic Characteristics, Revision 11*. 2018, NASA Socioeconomic Data and Applications Center (SEDAC): Palisades, NY.
36. World Bank. *Worldwide Governance Indicators*. 2018; Available from: https://info.worldbank.org/governance/wgi/.
37. Rico, M., et al., *Reported tailings dam failures. A review of the European incidents in the worldwide context.* Journal of Hazardous Materials, 2008. **152**(2): p. 846-852.
38. LPSDP, *Tailings Management - Leading Practice Sustainable Development Program for the Mining Industry*. 2016, Australian Government: Canberra, Australia.
39. WISE. *Chronology of major tailings dam failures*. 2020 3rd February 2019]; Available from: http://www.wise-uranium.org/mdaf.html.
40. Azam, S. and Q. Li, *Tailings Dam Failures: A Review of the Last One Hundred Years.* Geotechnical News, 2010.
41. Balabanova, B., et al., *Characterisation of heavy metals in lichen species Hypogymnia physodes and Evernia prunastri due to biomonitoring of air pollution in the vicinity of copper mine.* International Journal of Environmental Research, 2012. **6**(3): p. 779-794.
