# Supplementary material for: The social and environmental complexities of extracting energy transition metals
Source: Nat Commun. 2020 Sep 24;11:4823. doi: 10.1038/s41467-020-18661-9 (PMC7519138; doi:10.1038/s41467-020-18661-9)
Supplement: Supplementary file 3 — Description of Additional Supplementary Files [file 41467_2020_18661_MOESM3_ESM.pdf]

Description of Additional Supplementary Files

File Name: Supplementary Data 1
Description: List of public datasets used with download links and descriptions
