# Supplementary material for: The social and environmental complexities of extracting energy transition metals
Source: Nat Commun. 2020 Sep 24;11:4823. doi: 10.1038/s41467-020-18661-9 (PMC7519138; doi:10.1038/s41467-020-18661-9)
Supplement: Supplementary file 5 — Reporting Summary [file 41467_2020_18661_MOESM5_ESM.pdf]

nature research

Corresponding author(s): Eleonore Lebre

Last updated by author(s): Aug 19, 2020

Reporting Summary

Nature Research wishes to improve the reproducibility of the work that we publish. This form provides structure for consistency and transparency in reporting. For further information on Nature Research policies, see our Editorial Policies and the Editorial Policy Checklist.

Statistics

For all statistical analyses, confirm that the following items are present in the figure legend, table legend, main text, or Methods section.

n/a	Confirmed	
☐	☒	The exact sample size (n) for each experimental group/condition, given as a discrete number and unit of measurement
☒	☐	A statement on whether measurements were taken from distinct samples or whether the same sample was measured repeatedly
☒	☐	The statistical test(s) used AND whether they are one- or two-sided<br>*Only common tests should be described solely by name; describe more complex techniques in the Methods section.*
☒	☐	A description of all covariates tested
☒	☐	A description of any assumptions or corrections, such as tests of normality and adjustment for multiple comparisons
☐	☒	A full description of the statistical parameters including central tendency (e.g. means) or other basic estimates (e.g. regression coefficient) AND variation (e.g. standard deviation) or associated estimates of uncertainty (e.g. confidence intervals)
☒	☐	For null hypothesis testing, the test statistic (e.g. F, t, r) with confidence intervals, effect sizes, degrees of freedom and P value noted<br>*Give P values as exact values whenever suitable.*
☒	☐	For Bayesian analysis, information on the choice of priors and Markov chain Monte Carlo settings
☒	☐	For hierarchical and complex designs, identification of the appropriate level for tests and full reporting of outcomes
☒	☐	Estimates of effect sizes (e.g. Cohen's d, Pearson's r), indicating how they were calculated

Our web collection on statistics for biologists contains articles on many of the points above.

Software and code

Policy information about availability of computer code

Data collection	No software was used
Data analysis	No custom algorithms were required

For manuscripts utilizing custom algorithms or software that are central to the research but not yet described in published literature, software must be made available to editors and reviewers. We strongly encourage code deposition in a community repository (e.g. GitHub). See the Nature Research guidelines for submitting code & software for further information.

Data

Policy information about availability of data

All manuscripts must include a data availability statement. This statement should provide the following information, where applicable:

- Accession codes, unique identifiers, or web links for publicly available datasets
- A list of figures that have associated raw data
- A description of any restrictions on data availability

Download links and description of all publicly-available datasets used for this study are provided in the Supplementary Data file. The source data underlying Figs. 1 and 2 are provided in the Source Data file. Due to their proprietary nature, mining project data are only available from the corresponding author upon reasonable request.

Field-specific reporting

Please select the one below that is the best fit for your research. If you are not sure, read the appropriate sections before making your selection.

☐ Life sciences ☐ Behavioural & social sciences ☒ Ecological, evolutionary & environmental sciences

For a reference copy of the document with all sections, see nature.com/documents/nr-reporting-summary-flat.pdf

Ecological, evolutionary & environmental sciences study design

All studies must disclose on these points even when the disclosure is negative.

Study description	The study extracts values from 24 spatial variables at the location of 6,888 mining projects, and groups these values into 7 environmental, social and governance risk dimensions. Mining projects are then grouped by resources to obtain a risk profile for each of the 9 metal commodities studied. The second part of the study focuses on the geographic distribution of risks and resources and identifies key countries. The study uses existing data and does not generate new data.
Research sample	Mining project data including latitude and longitude were extracted from the S&P Global Market Intelligence database, which is a commercial database that gathers public disclosure data, mainly reported by project owners. The 6,888 mining projects represent all records from the database that are i) either operational or past the early exploration stages, and ii) have declared reserves and resources for the metal commodities analysed. The 24 variables are compiled from 24 publicly available global datasets from 14 different sources including the United Nations, the World Bank, the World Resources Institute, the NASA Socioeconomic Data and Applications Centre, the European Commission Joint Research Centre, the Japan Aerospace Exploration Agency, Birdlife International, Conservation International and several academic sources.
Sampling strategy	Not relevant. The mining project sample is the maximum possible sample, and its size is limited by global data availability. The number of datasets chosen for the analysis also depends on the availability of global-level data and its ability to accurately characterize the environmental, social and governance aspects analysed. The number of datasets is not a criteria for accuracy.
Data collection	Mining project data was extracted from the S&P Global Market Intelligence directly to an Excel file using the S&P database screener and Excel add-in. Spatial datasets were downloaded directly from their online repositories. Data was collected by the first author.
Timing and spatial scale	All data were collected at the beginning of the research project in 2019. Mining project data was extracted once from the S&P Global Market Intelligence in May 2019. The most recent versions for all spatial datasets used were downloaded individually between May and August 2019. All datasets used are global datasets, with varying resolution levels.
Data exclusions	The mining project sample excludes projects in early exploration stages (i.e. projects listed as "grassroots" and "exploration" in the database) on the basis that the development of these projects is subject to very high uncertainties, whereas projects in later stages of development are likely to be brought into production. Our global sample of operational and late stage projects is selected to be representative of the future global supply of energy transition metals. The same exclusion criteria was used in previous publications (Valenta et al. 2019, Lebre et al. 2019).
Reproducibility	Not relevant. The study can be reproduced using the same existing data collected by the authors and following the same methodological steps detailed in the methods section and in the supplementary information. The study does not generate new data.
Randomization	The study uses randomization twice for a sensitivity analysis and completeness test. 1) Sensitivity analysis for the risk matrix (Supplementary Figure 11) tests the stability of each risk dimension and of the total risk score. The test is to deliberately skew each risk dimension by raising them to a random power between 0.5 and 2, running this for 100 trials, using different sets of random numbers for each trial. 2) Completeness test (Supplementary Figure 12) re-runs the analysis 100 times, each time with a different - randomly selected - subset of the mining project sample. Subsets used represent 90% of the complete set (6199 projects).
Blinding	Blinding is not relevant to the study because it does not involve any participant and does not rely on possible differential treatment or assessments of outcomes

Did the study involve field work? ☐ Yes ☒ No

Reporting for specific materials, systems and methods

We require information from authors about some types of materials, experimental systems and methods used in many studies. Here, indicate whether each material, system or method listed is relevant to your study. If you are not sure if a list item applies to your research, read the appropriate section before selecting a response.

Materials & experimental systems

n/a	Involved in the study
☒	☐ Antibodies
☒	☐ Eukaryotic cell lines
☒	☐ Palaeontology and archaeology
☒	☐ Animals and other organisms
☒	☐ Human research participants
☒	☐ Clinical data
☒	☐ Dual use research of concern

Methods

n/a	Involved in the study
☒	☐ ChIP-seq
☒	☐ Flow cytometry
☒	☐ MRI-based neuroimaging
